# Influenza: National Trends Using the National Inpatient Sample Database from 1993 to 2015

**DOI:** 10.7759/cureus.7684

**Published:** 2020-04-16

**Authors:** Abdulrahman S Museedi, Mouhamed Nashawi, Abdullah Ghali, Aws Alameri, Abbas Alshami, Robert Nathanson

**Affiliations:** 1 Internal Medicine, University of Texas Health Science Center at San Antonio, San Antonio, USA; 2 Surgery, University of Texas Health Science Center at San Antonio, San Antonio, USA; 3 Internal Medicine, Jersey Shore University Medical Center, Neptune, USA

**Keywords:** influenza, national trends, cost effectiveness, mortality, discharge, flu

## Abstract

Background

There is a significant impact of influenza on individuals, families, and societies both economically and clinically. This significant impact is a result of hospital admissions, medication expenses, side effects, secondary bacterial infections, and more days off from work or other forms of reduced productivity for the patients or their caretakers. Our objective is to present the trends in the rate of hospital discharges per 100,000 population from the years 1993 through 2015, the mean age, and the inpatient mortality rate.

Methods

This is a retrospective study utilizing the National Inpatient Sample (NIS) from 1993 through 2015. Discharges due to influenza from 1993 to 2015 were identified, and the rate of discharges per 100,000 population, inpatient mortality, and mean age of discharged patients were trended. Linear regression was used to assess if the deviation from horizontal was statistically significant for the trends of the rate of discharges per 100,000 population, mean age, and percentage of the inpatient mortality.

Result

The mean age and inpatient mortality vary from year to year. The linear regression analysis for the trends was not statistically significant, and for the percentage of the inpatient mortality, the deviation from horizontal was not significant, P-value 0.75 and F-value: 0.09. Similarly, for the mean age, the deviation from horizontal was not significant with a P-value of 0.97 and an F-value of 0.001. However, the linear regression analysis for the rate of discharges per 100,000 population was remarkable for a statistically significant deviation from the horizontal with a P-value of 0.0002 and an F value of 19.5.

Conclusion

Recent advancements in influenza detection have made the detection feasible, quick, and cost-effective. However, the role of these advanced modalities on the outcome is still controversial. Our analysis revealed a significant increase in the rate of discharges due to influenza, but there was no significant change in the parentage of the inpatient mortality over the years between 1993 - 2015. Advanced influenza virus detection tests are now recommended in both outpatient (including emergency department) and inpatient admissions. The recent increase in inpatient admissions could be due to better detection modalities. However, no change in the percentage of inpatient mortality makes the impact of these detection tests on the outcome questionable. A further prospective study is warranted to assess the impact of these tests on the outcome.

## Introduction

Influenza, known colloquially as “the flu,” is a viral infection of the influenza virus with ramifications that range from relatively benign to fatal on the scale of the patient. Common symptoms are very broad, sharing an overlap with a wide array of infectious diseases and inherent pathologies, such as malaise, fever, coughs, and myalgias [1]. While usually self-limited and benign, influenza may also manifest with more severe sequelae that warrant prompt medical management, such as lung injury, myocarditis, cardiomyopathy, and even neurological dysfunction in the form of febrile seizures, encephalitis, or peripheral neuropathies, such as Guillain-Barré syndrome [2-6]. On an epidemiological scale, influenza has been notorious to cause outbreaks with significant morbidity and mortality, such as the 2009 influenza pandemic, estimated to have had a global mortality count of up to 500,000, with comparatively more years of life lost relative to other flu seasons due to the higher proportion of younger deaths [7]. In the United States (US), annual hospitalizations secondary to influenza range from around 140,000 to over 700,000 based on predictive models and records from healthcare institutions [8]. This represents a significant multifactorial impact on society. For example, the economic and clinical consequences of influenza-related hospitalizations include days of work or school missed, mitigated productivity of affected patients, the burden of caregivers, medication expenses, treatment complications, and secondary illnesses that result from an immunocompromised status. While complex to forecast, attempts in the literature to gauge the fiscal impact of influenza in the US estimate the figure to be in the range of $45B USD to as much as $87B, depending on the analyses and factors weighted [9-10]. Nevertheless, there is a profound effect of influenza on the social framework in the United States, namely, within healthcare institutions [11]. To understand this effect in the context of health outcomes and clinical decision-making, it is prudent to assess how hospital discharges related to influenza have changed with advances in healthcare. In this work, we seek to present trends in the rates of hospital discharges within the population from the years 1993 through 2015, as well as to characterize demographic variables (such as age) and outcome variables (such as inpatient mortality rates). Doing so will present the opportunity to report shifts in influenza treatments, as well as to elucidate areas for improvement on the institutional level.

## Materials and methods

Our analyses are composed of a retrospective study utilizing The National Inpatient Sample (NIS) from 1993 through 2015. The NIS is a database that encompasses inpatient healthcare data utilized by policymakers and academics alike to trend healthcare utilization, outcomes, and changes in empirical structures within the architecture of the United States healthcare system [12]. Discharges due to influenza from 1993 to 2015 were identified by using ICD-9 codes sorted by Clinical Classification Software (CCS) [13]. The rate of discharges per 100,000 in the population, inpatient mortality, and mean age were trended along the duration mentioned. Linear regression was then utilized to assess if the deviation from horizontal was statistically significant for the rate of discharges per 100,000 population, mean age, and percentage of the inpatient mortality.

## Results

**Figure 1:**
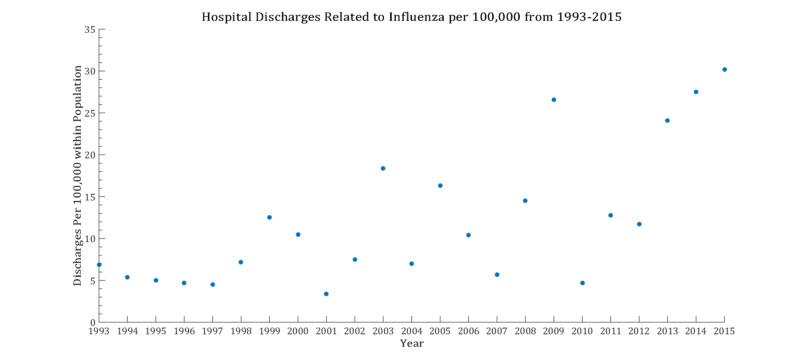
Hospital discharges for influenza-related admissions from 1993-2015

**Figure 2:**
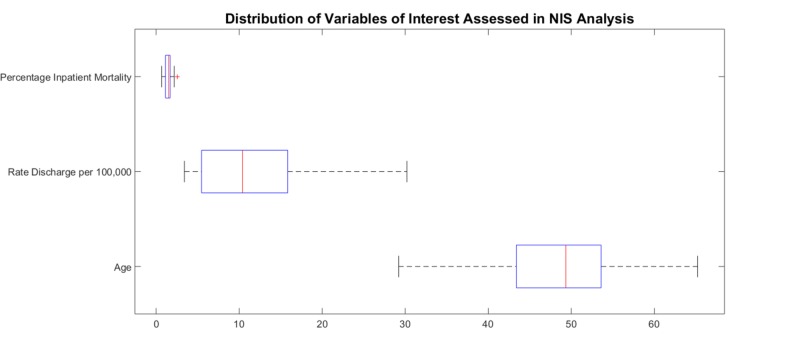
Characterization of variables in influenza discharge analysis NIS: National Inpatient Sample

**Figure 3:**
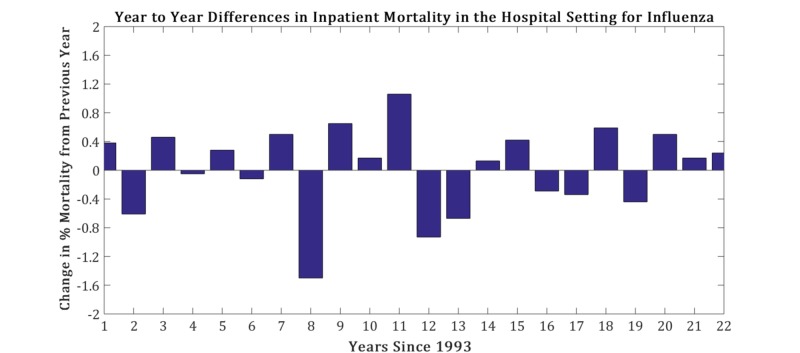
Yearly inpatient mortality differential in patients hospitalized with influenza

**Figure 4:**
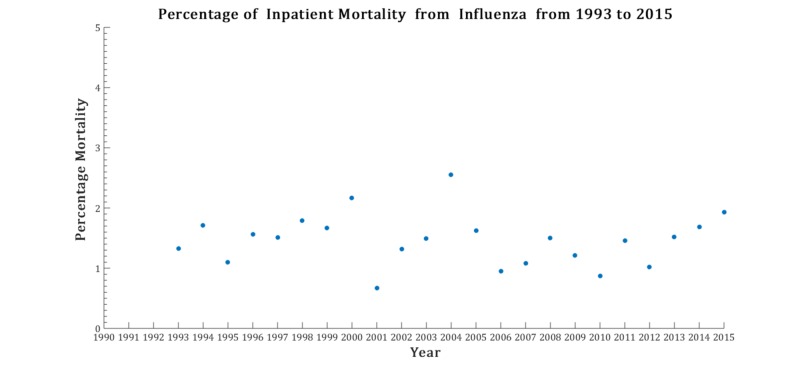
Yearly inpatient mortality in patients hospitalized with influenza from 1993 to 2015

The mean age, inpatient mortality, and discharge rates vary from year to year. Influenza discharges over the time interval are found in Figure 1. The characterization of the variables are listed in Figure 2. For the percentage of inpatient mortality, the deviation from the linear configuration was not statistically significant, with a P-value = 0.75 and F-value = 0.09. Similarly, the mean age was also not statistically significant, with a P-value = 0.97 and F-value = 0.001. However, the linear regression analysis for the rate of discharges was remarkable for statistical significance with a P-value = 0.0002, and F-value = 19.5. No pattern was discernable in year-to-year changes in inpatient mortality (taken by subtracting the mortality percentage of consecutive years) as noted in Figure 3. The magnitude of inpatient mortality from year-to-year did not change significantly and did not exhibit a consistent pattern as illustrated by Figure 4.

## Discussion

Recent advancements in the detection of influenza make surveillance of the virus feasible, quick, and cost-effective in the clinical setting [14-16]. Moreover, there has been recent interest in the public health sector in mining online data to effectively predict local changes in influenza flux to predict epidemics of the virus in an attempt to control outbreaks [17]. However, the role of these methods on end outcomes is still controversial. Our analysis has revealed a significant increase in the rate of discharges due to influenza but no significant changes in the percentage of inpatient mortality over the years between 1993 to 2015. The latter phenomena are intact in the setting of varying mean age of admissions throughout the years analyzed. Advanced influenza virus detection tests are now recommended in both outpatient (including emergency departments) and inpatient admissions, and vaccination against influenza is still encouraged and supported by many organizational bodies, whether it be in education, the private sector, and government, depending on the roles of individuals at these institutions. It is noted that there was a recent increase in the inpatient admission counts from influenza as extrapolated by a marked increase in hospital discharges related to influenza. The interval 2013 to 2015 was comparable to the 2009 influenza pandemic year in the discharge rate for influenza. This increase in discharges may have been due to more efficient detection modalities, resulting in more influenza diagnoses within a population. However, the lack of change in the percentage of the inpatient mortality year-to-year makes the impact of these detection tests on the end-outcome for the respective admission questionable. There were no more than two consecutive negative values in Figure 3, noting that there were no more than two consecutive decreases in mortality related to influenza.

**Figure 5:**
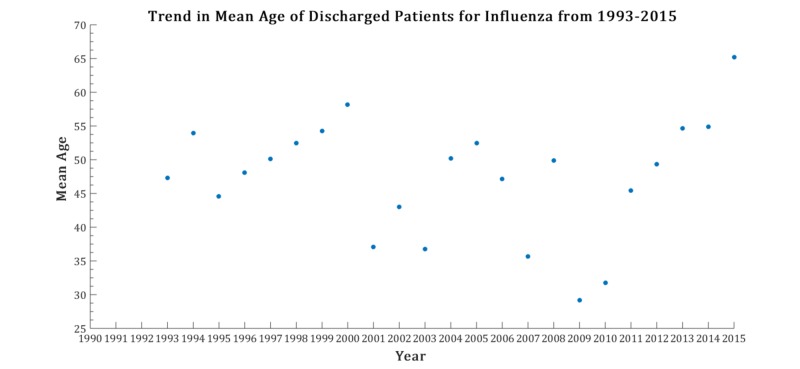
Yearly mean age in patients hospitalized with influenza from 1993 to 2015

In the context of increased discharges, stagnant inpatient mortality raises the question if the discharges were due to a higher turnover of the same capability to manage influenza by healthcare institutions. A further prospective study is warranted to assess the impact of these tests on health outcomes. Furthermore, while detection methods have been bolstered by advances in technology and implementation, the magnitude of the mortality percentage number has remained relatively stagnant over nearly two decades, representing a possible deficiency in institutions and providers to be equipped with better decision-making algorithms to effectively manage the patients with influenza. Another area of potential interesting further study and analysis is the trend that while hospital discharge rates are increasing in recent times, so is the mean age of hospital discharges with influenza since the 2009 influenza pandemic, which shows not only a return to baseline rates of discharges but the first yearly mean age greater than 65 years of age (Figure 5). This may represent challenges primary care institutions have at reaching out to older populations in vaccination efforts, preventative measures, and health education. Alternatively, this may represent longer life expectancies reached with better healthcare initiatives and longer living patients perhaps contributing to the discharge population for influenza, driving the mean figure up. More research needs to be done to characterize the features of patients who undergo hospitalization and subsequent discharge for influenza with a focus on demographics, socioeconomic status, locale, and clinical workup to better identify areas of improvement.

## Conclusions

Our analysis revealed a significant increase in the rate of discharges due to influenza but there was no significant change in the parentage of the inpatient mortality over the years between 1993 and 2015 which makes the role of the advanced detection modalities on the outcome controversial. This is study is limited by being retrospective, and further prospective studies are warranted to assess the impact of these tests on the outcome. 
